# Development of High Hydrostatic Pressure Applied in Pathogen Inactivation for Plasma

**DOI:** 10.1371/journal.pone.0161775

**Published:** 2016-08-25

**Authors:** Chunhui Yang, Guohui Bian, Hong Yang, Xinmin Zhang, Limin Chen, Jingxing Wang

**Affiliations:** 1 Institute of Blood Transfusion, Chinese Academy of Medical Sciences (CAMS) & Peking Union Medical College (PUMC), Chengdu, Sichuan, China; 2 Zhengzhou Feilong medic devices Co., Ltd, Zhengzhou, Henan, China; GERMANY

## Abstract

High hydrostatic pressure has been used to inactivate pathogens in foods for decades. There is a great potential to adapt this technology to inactivate pathogens in plasma and derivatives. To better evaluate the potential of this method, pathogen inoculated plasma samples were pressurized under different pressure application modes and temperatures. The inactivation efficacy of pathogens and activities of plasma proteins were monitored after treatment. The CFUs of *E*.*coli* was examined as the indicator of the inactivation efficiency. The factor V and VIII were chosen as the indicator of the plasma function. Preliminary experiments identified optimized treatment conditions: 200-250MPa, with 5×1 minute multi-pulsed high pressure at near 0°C (ice-water bath). Under this conditions, the inactivation efficacy of EMCV was >8.5log. The CFUs of *E*. *coli* were reduced by 7.5log, *B*. *cereus* were 8log. However, PPV and *S*. *aureus* cannot be inactivated efficiently. The activities of factor II, VII, IX, X, XI, XII, fibrinogen, IgG, IgM stayed over 95% compared to untreated. Factor V and VIII activity was maintained at 46–63% and 77–82%, respectively.

## Introduction

Blood transfusion can result in transfusion transmitted infection disease (TTID). In the last decades, the strategies of donor selection and specific testing have been implemented to reduce this risk. The effect is obvious, especially after the introduction of nucleic acid testing (NAT) [1]. It has minimized the “window period” and reduced the risk of transfusion transmitted virus infections. However, the risk of TTID still exists because: when the virus infection is below a certain level, the screening methods are unable to detect it, and errors may occur during the detection process; The continued new emergence or reemergence of pathogens threatened blood safety and the intervals between the first recognition of transfusion risk and the implementation of a preventive strategy can be several years [2]. As an additional safety measure, pathogen reduction technology (PRT) can be an important tool to further reduce the risk of TTID in some blood components and has been implemented by some blood centers throughout the world.

Based on different operating principles, PRTs can be broadly classified into three categories: physical (heat treatment, nanofiltration), chemical (solvent detergent S/D) and photochemical (methylene blue MB treatment, riboflavin-based and psoralen-based treatment). Every method has its limitations [3].

High hydrostatic pressure (HHP) technology, also known as high pressure processing or ultra-high pressure, is a physical pathogen reduction technology in which high pressure was applied through fluids on objects [4]. It was firstly used in food preservation in the early 1990s [5]which increased the shelf life of products such as milk, fruit, meat, etc. Because of its potential advantages such as no chemical additive introduced into samples, ease of operation, short treatment time and high throughput capability, HHP might also be a promising tool in the decontamination of biologic therapeutic products such as vaccines, animal serum and blood products. However, the procedure of HHP used in the food industry can not be used for blood products because maintaining the function of blood proteins is essential. Although some previous studies alluded to the possibility of using HHP for pathogen reduction in plasma [6, 7], these studies were restricted to limited pathogens and plasma proteins. To better evaluate the potential of this method, we optimized HHP based on a preliminary assessment of bacterial inactivation and maintenance of two labile plasma factor coagulation activities, factor V and factor VIII. We subsequently utilized the optimal conditions to evaluate several model viruses, bacteria, and an array of coagulation factor activities.

## Material and Methods

### Bacterial and virus species

The following bacterial were obtained from Sichuan Institute of antibiotics: *Escherichia coli* (*E*. *coli* SIIA0726 gram+), *Bacillus cereus* (*B*. *cereus* SIIA0554 gram-), *Staphylococcus aureus* (*S*. *aureus* SIIA2260 gram+) and *Pseudomonas aeruginosa* (*P*. *aeruginosa* SIIA2265 gram-). The bacteria are chosen according to the bloodstream infection report in China Ministry of Health National Antimicrobial Resistant Investigation Net, Mohnarin, [8] including gram positive and negative strain, coccus and bacillus. The model viruses were recommended by ATCC and CCTCC: Pseudorabies virus (PRV, CCTCC GDV131) and encephalomyocarditis virus (EMCV, ATCC VR-129B) were assessed in Vero cells. The bovine viral diarrhea virus (BVDV, ATCC VR-1422) was assessed in Bovine Turbinate (BT) cells. The porcine parvovirus (PPV, ATCC VR-742) was assessed in swine testis (ST) cells. Four model viruses represent several common transfusion transmitted viruses. The detailed features of model viruses are showed in Table 1.

**Table 1:** Features of model viruses.

Virus	Strand	Nucleic acid	Lipid envelop	Model virus for
EMCV	ss	RNA	No	HAV
PRV	ds	DNA	Yes	HBV
BVDV	ss	RNA	Yes	HCV
PPV	ss	DNA	No	HPV B19

### Preparation of plasma samples

Apheresis fresh frozen plasma was provided by Guanghan plasma station and stored at -30°C until use. The plasma was thawed in a 37°C water bath and 4.5mL aliquots were transferred to plasma bag. The bacterial and virus suspension was mixed with plasma aliquots by 1:10 dilution. The average initial titer for bacterial was 6-9Log CFUs per mL, and for virus is 8-11Log. The control samples were prepared by replacing bacterial and virus suspension with an equal volume of culture medium.

### HHP treatment

The HHP device was provided by Zhengzhou Feilong Medic device Co., Ltd (Zhengzhou, China). It was consisted of a high pressure chamber, an electric power driven oil pump and a pressure gauge. The plasma sample size was 5mL and stored in a plasma bag specially made by Suzhou Laishi blood transfusion equipment Co., LTD. Samples were placed into the chamber filled with water as the pressure transmitting medium. The pressure level was driven by the pump. Two treatment temperatures were used: room temperature and 0°C. To decrease the temperature, the transmitting medium was replaced with a mixture of ice and water between every treatment. The application mode of HHP treatment is continuous or cycling. In the continuous mode, the device compressed to a target pressure, then held at that pressure for a specified time before decompression to atmospheric pressure (single-pulsed HHP, [spHHP]). In the cycle mode, the treatment was divided into different cycles (multi-pulsed HHP, [mpHHP]). In a cycle, the pressure was increased to setting level, holding for 1 minute, decompression, and then followed by another cycle.

### Assessment of pathogen reduction efficacy

Viral samples were determined by endpoint titration in micro titer plate assays (1:10 serial dilution, eight parallel samples per dilution) before and after HHP treatment. Host cells were cultured with viral samples and incubated at 37°C with 5% CO2 for 7 days. The cytopathological effects were inspected by microscope daily. Results were calculated by the Karber Method and expressed as log_10_ of tissue culture infection dose (TCID_50_). [9]

The detection of bacterial was calculated by colony-forming units (CFUs). Aliquots (1 mL) of the samples and 1:10 serial dilutions thereof were plated on agar and incubated at 37°C. After 24 hours incubation, the CFUs were counted.

### Plasma proteins assays

The activities of plasma proteins were measured before and after HHP treatment. Levels of factor II, V, VII, VIII, IX, X, XI, and XII, and fibrinogen were analyzed by a sysmex CA-1500(sysmex, Kobe, Janpa) and matching deficient plasma samples. Total protein was measured by the biuret method (Shanghai Rongsheng kits). Total IgG, IgM, IgG1, IgG2, IgG3 and IgG4 were measured by an in-house Enzyme-linked immunosorbent assay using kits from Immunology Consultants Laboratory, Inc and the Invitrogen human IgG subclass profile Kits.

### Statistics

Each experiment was repeated at least twice and three independent experiments were performed to ensure the accuracy. Results were expressed by a graphing and analysis software package (GRAPHPAD PRISM, GraphPad Software, Inc., La Jolla, CA, USA). Two tailed paired t-tests were used to compare the difference between two given groups.

## Results

### Optimization of HHP treatment

**Fig 1:**
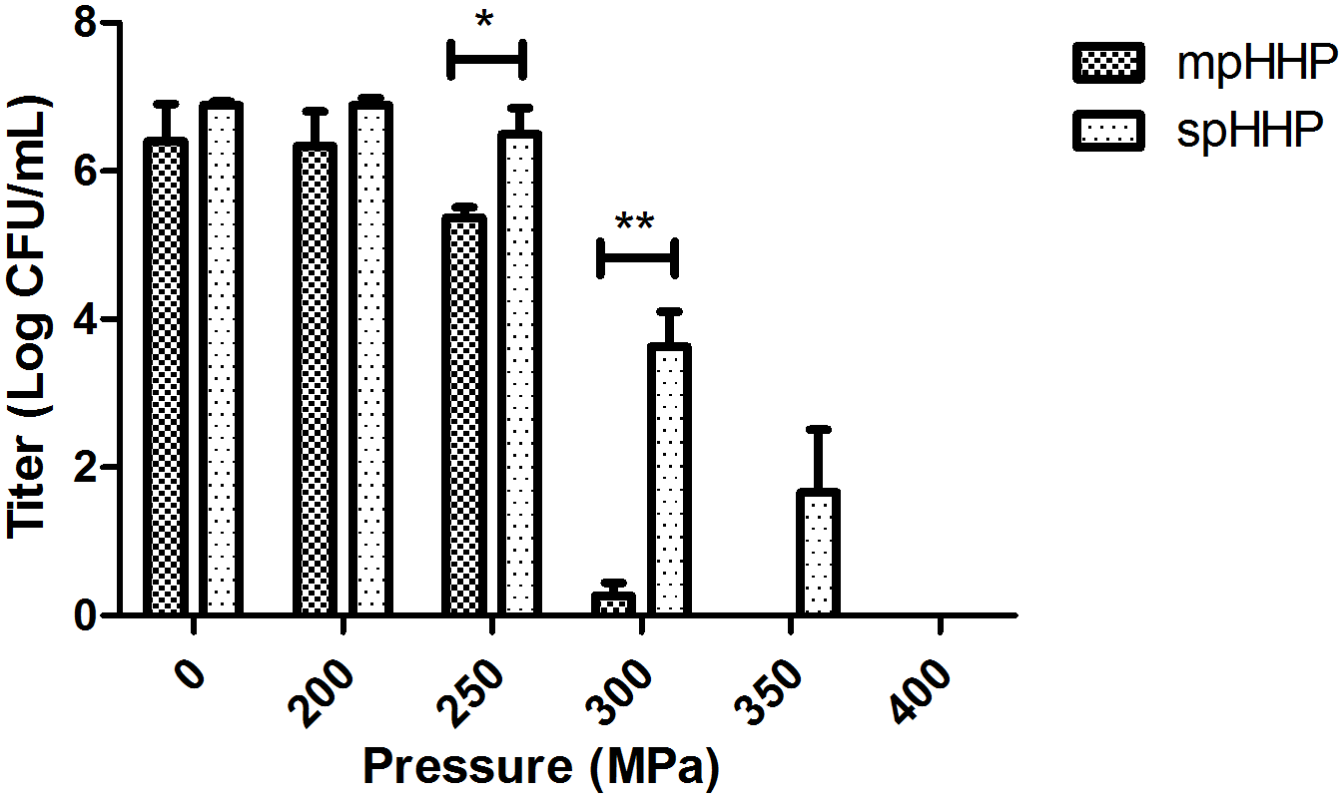
Inactivation of *E*.*coli* under mpHHP (5 cycles of 1 min) and spHHP (1 cycle of 5 min) application modes with escalated pressure, n = 3.

**Fig 2:**
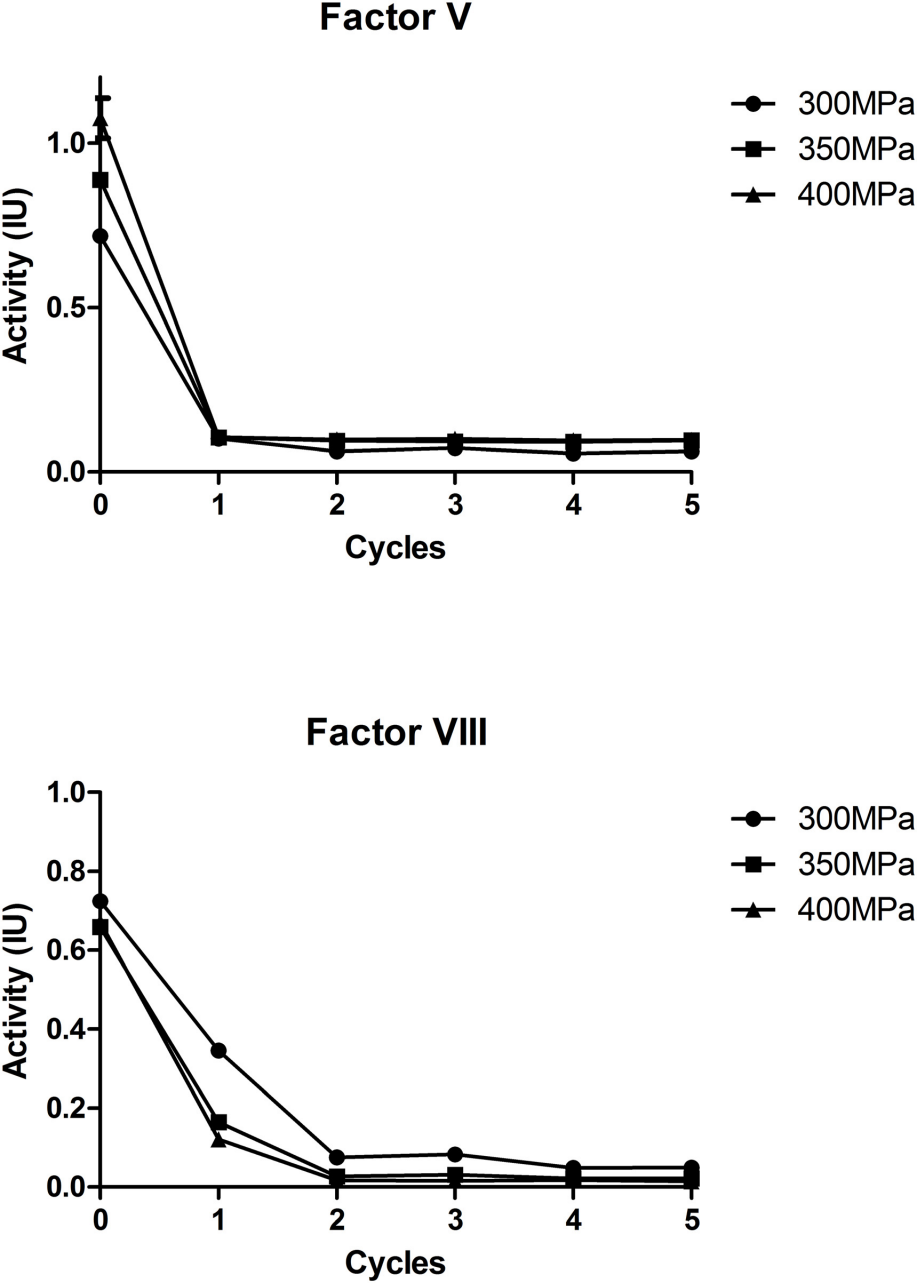
Change of activities of coagulation factor V and VIII under mpHHP treatments (5 cycles of 1 min) with escalated pressure (≥ 300MPa), n = 3.

HHP treatment has been regarded as an important innovation for food industry for decades [10]. Many basic food products like milk or fruits and even ethnic food products have been tested under pressurization [11]. However, the possibility of using HHP in blood products is still experimental. The greatest challenge is how to balance inactivation with maintenance of coagulation factor activity. In our study, it was optimized through two parameters: the temperature and HHP applied mode. The CFUs of *E*.*coli* was examined as the indicator of the inactivation efficiency. The factor V and factor VIII, two of the most unstable plasma proteins were chosen as the indicator of the plasma function. A suspension of *E*.*coli* in human plasma was treated under spHHP and mpHHP separately for 5 minutes at room temperature. We observed that under the spHHP mode, the *E*.*coli* could not be inactivated until the pressure level increased to 400MPa. In comparison, under the mpHHP mode, the reduction of *E*.*coli* began at 250MPa, was >5 log at 300MPa and no *E*.*coli* was detected at 350 or 400MPa (Fig 1). However, neither the spHHP nor mpHHP mode maintained the activity of factor V and VIII when the pressure level was over 300MPa (Fig 2). The activities greatly diminished after 1 min treatment. The result indicates that at the room temperature, both mpHHP and spHHP treatments have similar effect on factor V and VIII, but have different inactivation efficacy of *E*.*coli*.

For the HHP treatment, as the pressure level or treated time increases, the inactivation efficacy also increases. Previous reports found that higher reduction of *E*.*coli* in milk at 400MPa than 300MPa and higher reduction at 450MPa than 350MPa of *E*.*coli* in liquid whole egg [12]. This level of pressure (300-450MPa) was suitable for food industry, because of a lower requirement for maintaining protein function. However, coagulation plasma proteins are more sensitive to HHP. The changing of HHP application mode has been proved useful for inactivation. Bradley et al indicated that the repeated volume expansion and contraction with each pressure pulse may cause physical dissociation of viral subunits [6], increasing inactivation of bacteriophage lambda as much as an additional 2 log. Rivalain et al also demonstrated that cycling HHP treatment increased inactivation of *S*. *aureus* and the effect was further improved by increasing cycle numbers [7]. Various terms have been used to define mpHHP treatment [13–15]. Sencer summarized six typical reported mpHHP modes, and Donsì et al found that rather than adding pulse number combining pulse holding time and number of pulses contributed to efficiency of the mpHHP treatment [16]. In our current study, the treatment is consisted of successive spHHP. With five 1-miniute cycles using 300 MPa, inactivation of *E coli* increased >3 log compared to that of one 5-minute cycle (spHHP). Although the application of mpHHP has a better inactivation effect than spHHP, the activities of coagulation factor V and VIII were still severely compromised under this pressure level.

**Fig 3:**
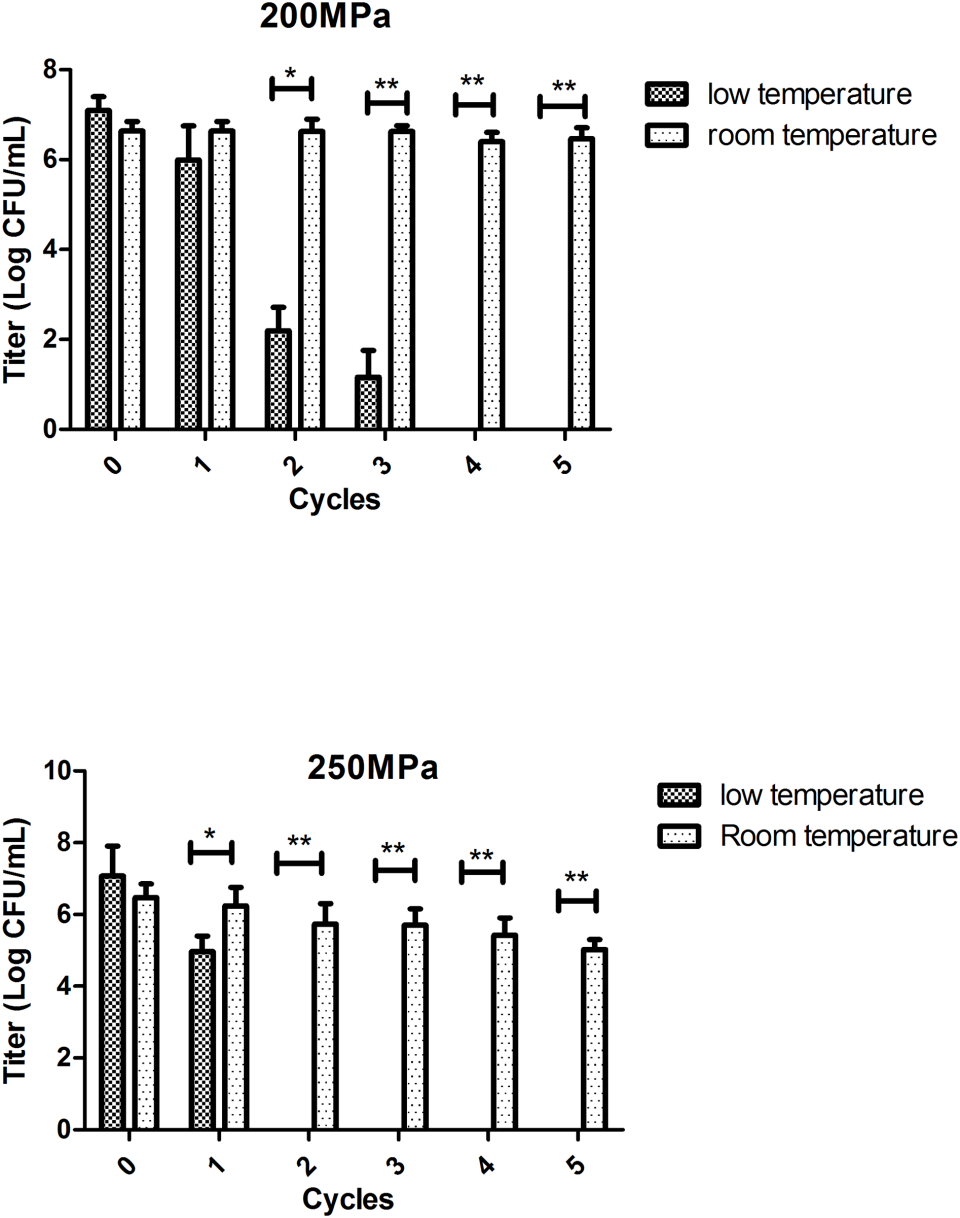
Inactivation of *E*.*coli* under two mpHHP modes (200/250 Mpa, 5 cycles of 1min) at low temperature (near 0°C) and room temperature, n = 3.

**Fig 4:**
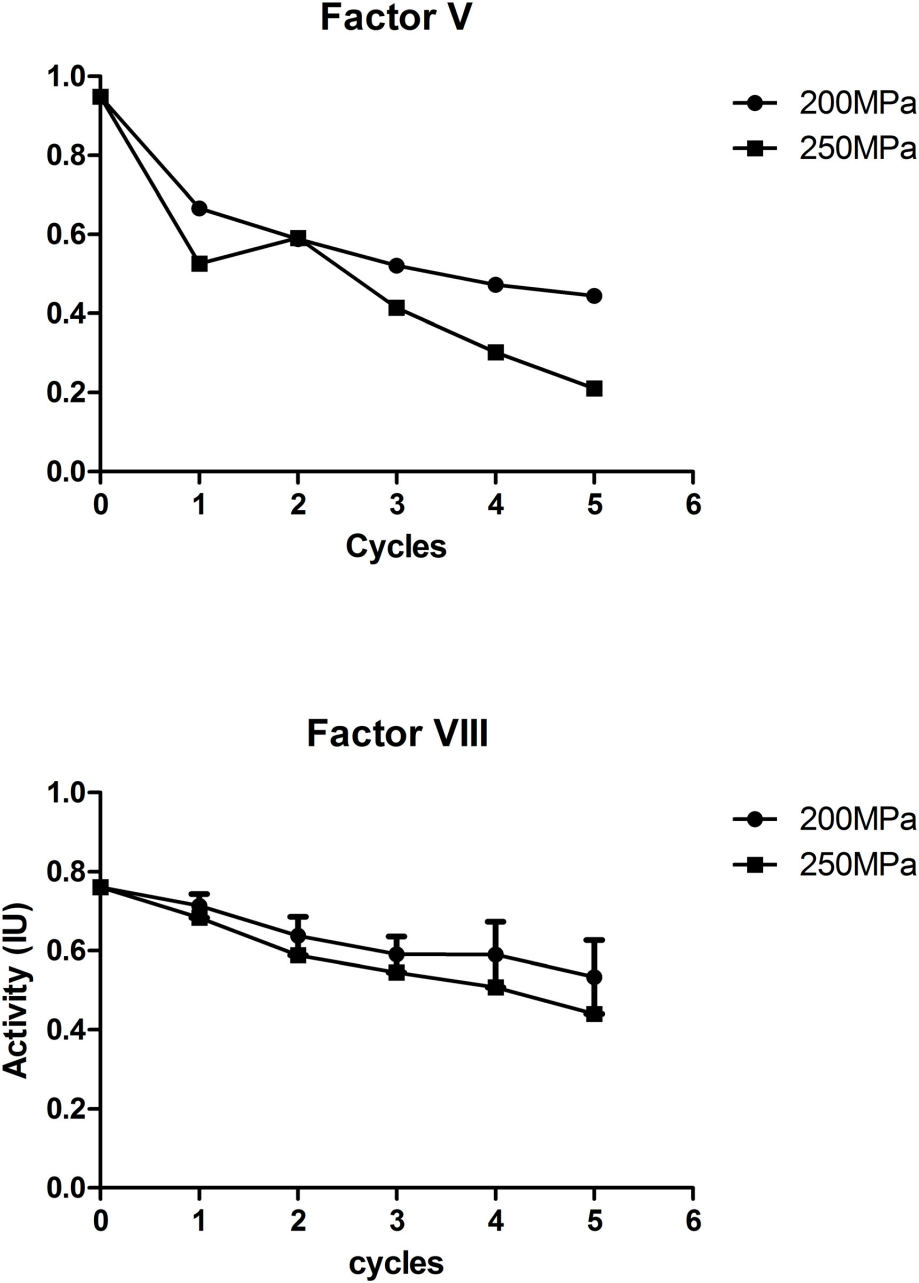
Activities of factor V and VIII after mpHHP treatment (200/250 Mpa, 5 cycles of 1min) at near 0°C temperature, n = 3.

Besides the application mode, temperature is another important parameter during the treatment. In food industry, a large range of temperature can be applied, but for plasma, only a low temperature is feasible. Previous studies reported that sub-zero temperature has a synergistic effect with pressure on pathogen inactivation [17], and that optimal inactivation of phage occurred between -10 to10°C [18]. Bradley et al reported that low temperatures can enhance bacterial inactivation by high pressure while maintaining protein activity [6]. To protect the function of coagulation factors, we combined the mpHHP treatment with a low temperature (near 0°C). It has not only decreased the effective pressure level to 200-250MPa, but also protected the activities of factors. Compared to room temperature, HHP at 0°C showed a better inactivation efficacy for *E*.*coli* (Fig 3). The titer of *E*.*coli* was obviously decreased after 2 cycles of mpHHP treatment and was undetectable after 4 or 5 cycles at 200 or 250MPa respectively. Under this test condition, activity of factor VIII was reduced by less than 20%. Although the activity of factor V was still severely compromised under low temperature conditions, the percent retention was higher than room temperature treatment (Fig 4). After 5 cycles 200MPa or 2 cycles 250MPa mpHHP treatment at low temperature, factor Vactivity still was > 50%.

### Effect of Optimized HHP treatment on different pathogens

**Fig 5:**
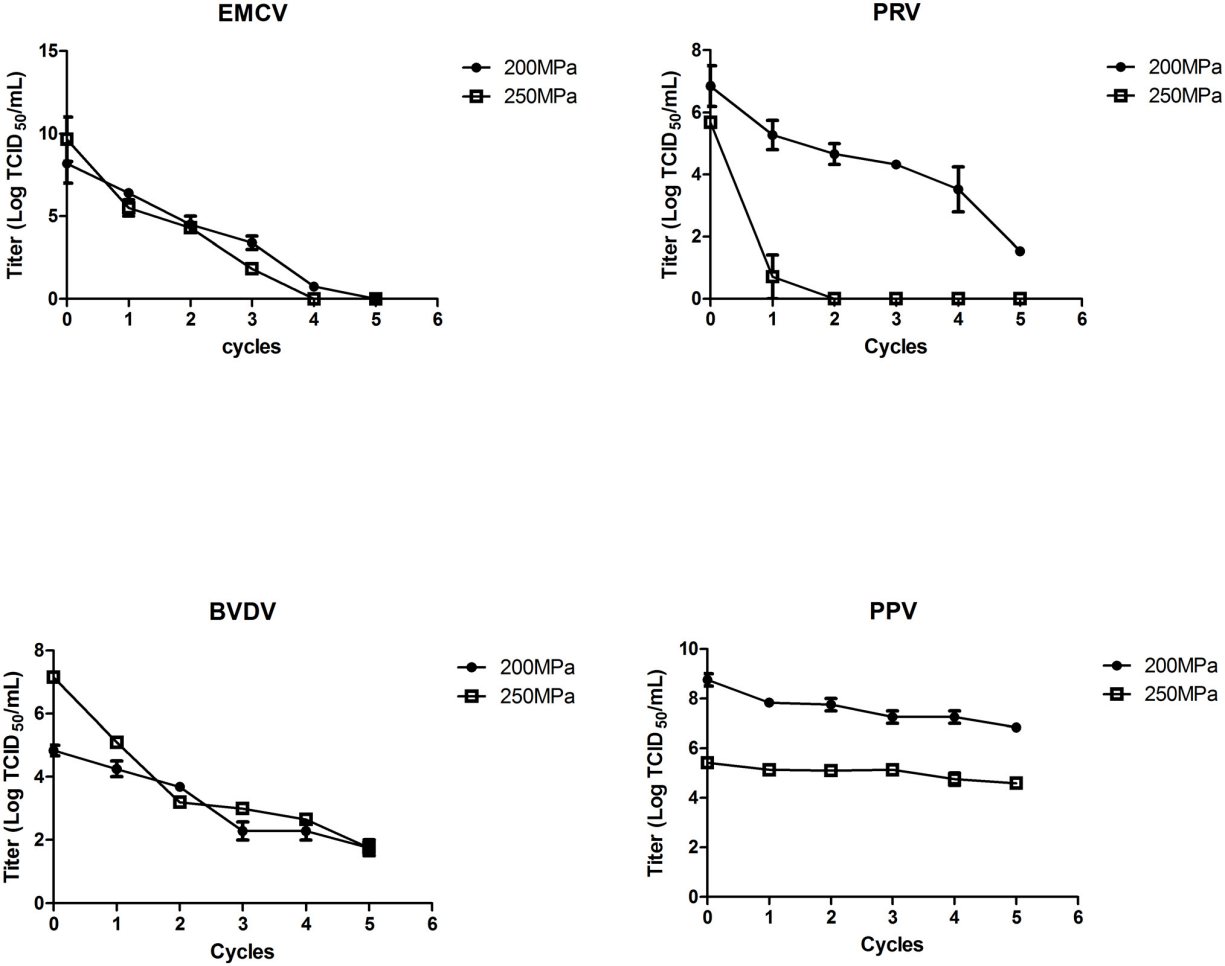
Kinetics of virus inactivation by optimized HHP treatment (200/250 Mpa, 5 cycles of 1min, at near 0°C temperature), n = 3.

According to our optimization study, to ensure the activities of coagulation factors, the pressure level was limited to 200-250MPa and the number of cycles should be less than 5 cycles of 1min for 200MPa and 3 cycles of 1 min for 250MPa. As previous studies only addressed the effect of HHP on limited number pathogens, we expanded the scope of our research to multiple representative blood-borne bacteria and viruses, in order to evaluate the general performance of mpHHP under low temperature. Under optimal conditions (200 MPa, 5 cycles or 250 MPa, 2 cycles), the effect of treatment on model viruses was summarized in Fig 5. Under the 200MPa pressure, 8.2log, 3.1log and 3.8log titer reduction of EMCV, BVDV and PRV was observed after 5 cycles. When the pressure level increased to 250MPa, 5.4log, 4log and 5.7log reduction of the three viruses was achieved after only 2 cycles of treatment. However PPV was resistant to these conditions and PPV titer was not reduced even after 5 cycles of treatment at 250 MPa.

The enveloped viruses, BVDV and PRV, were effectively inactivated. However, non-enveloped viruses showed different susceptibilities. EMCV can be inactivated easily under 200MPa pressure in 2 minutes, but PPV is insensitive to pressure. Hess et al found that 552MPa mpHHP can inactivate PPV [19], but in this condition, the plasma function can not be maintained. As a model virus for human parvovirus B19, PPV is a non-enveloped single-stranded DNA virus which is also less easily inactivated and show much lower log reductions than other blood PRTs [20, 21]. The character of small size and non-envelope with tightly interdigitated capsid proteins might be the reasons why PPV can survive from HHP and other treatments [22].

**Fig 6:**
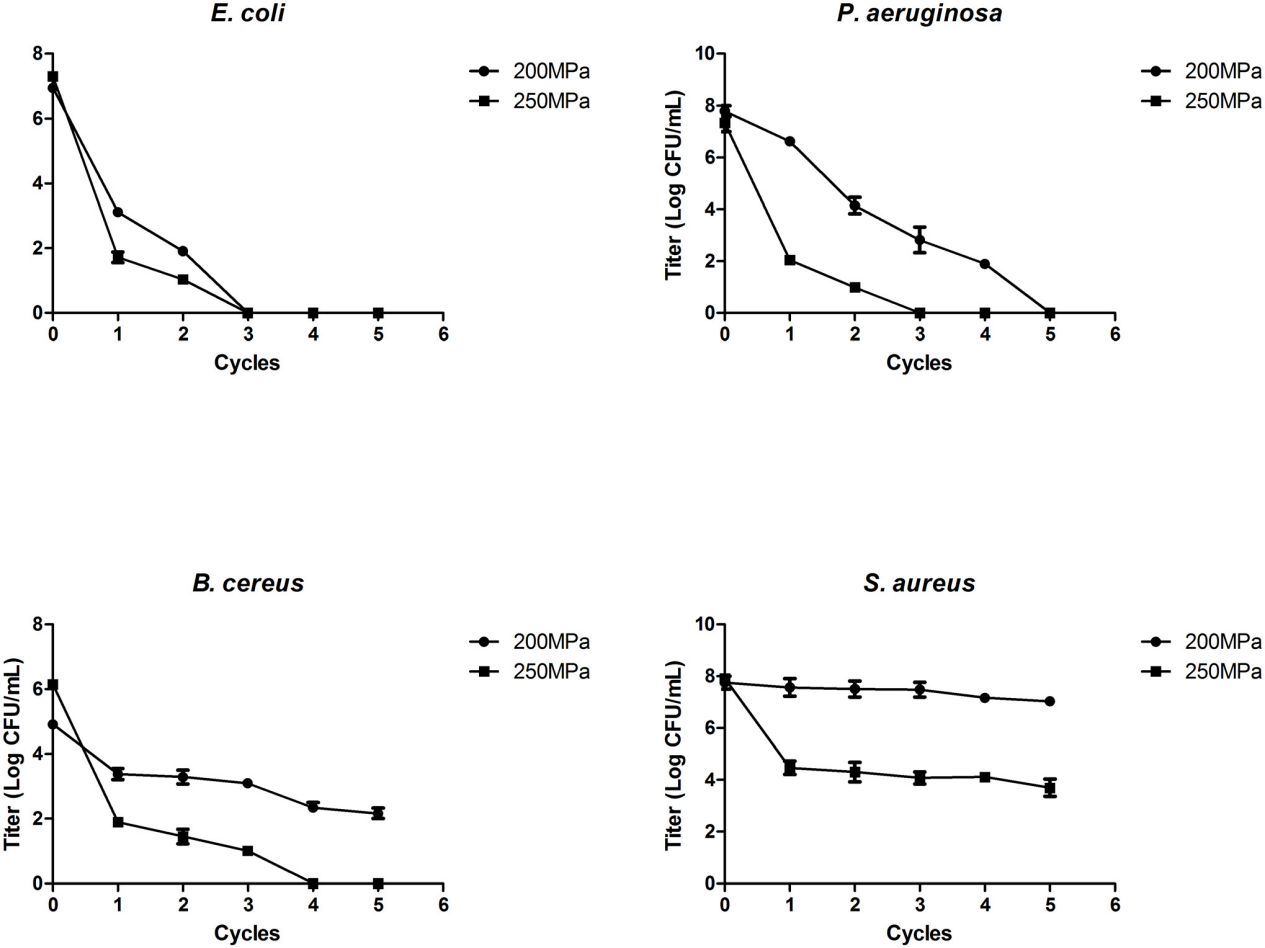
Kinetics of bacterium inactivation by optimized HHP treatment (200/250 Mpa, 5 cycles of 1min, at near 0°C temperature), n = 3.

Different bacteria also show different sensitivity to HHP treatment. The result of bacterial inactivation was showed in Fig 6. The *E*. *coli*, *P*. *aeruginosa* and *B*. *cereus* were able to be fully inactivated by 250MPa pressure after 4 cycles of treatment. Although the inactivation of *S aureus* was not significant at 200 MPa, approximately 4 log inactivation of *S aureus* was observed after 1 cycle at 250 MPa. For *B cereus*, bacterial count declined by 3.5 log after the first cycle and declined more slowly in subsequent cycles.

Our results revealed that the HHP treatment was more effective on Gram negative bacterial *E*.*coli* and *P*.*aeruginosa* than Gram positive bacterial *S*. *aureus* which only showed a slight reduction of CFU after optimized HHP treatment. Rivalain et al combined with a mpHHP model (300MPa, 5 cycle of 2min), a sub-zero temperature (-5°C) and a high PR (the speed to reach the target pressure, 50MPa/s) to inactivate *S*. *aureus* in plasma [7]. It led to a close to 8log reduction. However, the activities of plasma proteins haven’t been evaluated at the same time. Patterson discussed the efficacy of HHP on different microorganisms and pointed out the Gram-positive bacteria tend to be more resistant to pressure than Gram-negatives and cocci are more resistant than rod-shaped bacteria in general [23]. The complexity of cell membrane of each bacterium decides the degree of sensitivity to pressure. However, there are still some exceptions to the rules. In our research, the Gram positive bacterial *B*. *cereus* was significantly reduced even after a low pressure level treatment. Benito et al found certain strains of *E*. *coli* can be exceptionally pressure resistant [24]. Although the inactivation of Gram-positive bacteria is a challenge for HHP, the storage condition of plasma can be helpful. Storage in frozen made bacterial in plasma is hard to survive and proliferate. The reports of bacterial contamination are few [25]. We also found freezing plasma 2 hours after HHP treatment, the CFU of staphylococcus reduced up to 7log. Form above; bacterial inactivation in plasma is not the major problem.

### Influence of Optimized HHP treatment on plasma proteins

**Fig 7:**
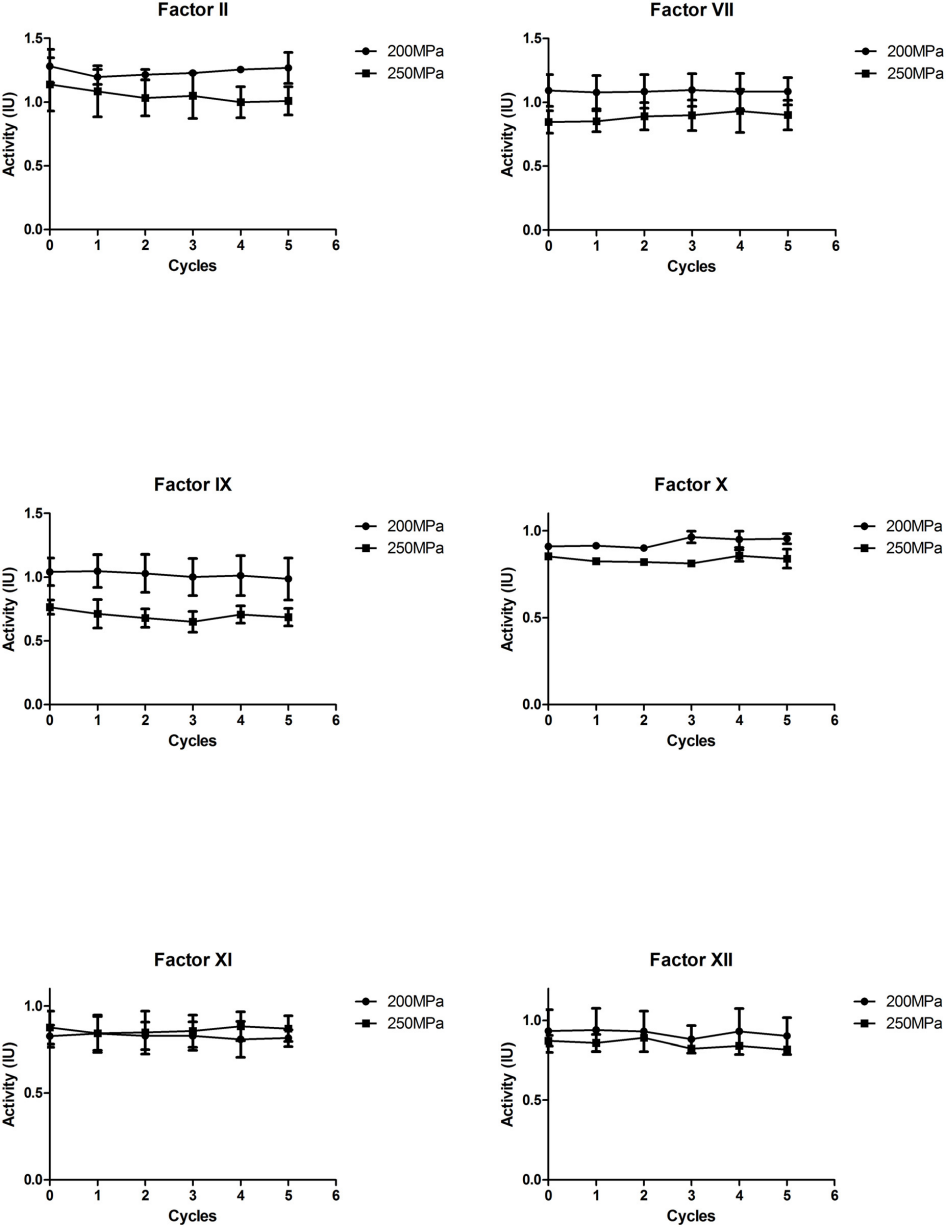
Activities of coagulation factors after optimized HHP treatment (200/250 Mpa, 5 cycles of 1min, at near 0°C temperature), n = 3.

Fig 7 shows the results of the activities of coagulation factors under the optimized HHP. The activities of factor II, VII, IX, X, XI and XII remain stable during the 5 cycles HHP treatment of 200MPa and 250MPa at near 0°C. The percentage retention of these factors was over 95%. The assessment results of total protein, fibrinogen and immune globulin also show the optimized HHP treatment did not affect the content of these proteins (Table 2). There are no difference between the control and HHP treated samples (P>0.05). A previous review article reported that most PRT results in a 20–30% loss of Factor VIII, while other coagulation factor activities are better retained [26]. According to Chinese guiding principles for blood product pathogen remove/inactivation, the pathogen reduction should be ≥ 4 Log and the activity of FVIII must≥ 0.5 IU/ml, the content of total protein≥ 50 g/L [27, 28]. In our study, factor V had an approximate 50% loss even with optimized treatment and it was the most susceptible factor under HHP treatment, which is in contrast to the fact that Factor VIII, an evaluation index in guidelines, is the most vulnerable by PRT treatment. This results indicated factor V should also be concerned in HHP treatment. Some plasma PRTs also affected other plasma proteins like MB treatment decreasing fibrinogen level and the S/D process resulting in a high loss of protein S [29, 30]. In our experiments, we did not find these damages.

**Table 2:** The content of plasma proteins after HHP treatment (200/250 Mpa, 5 cycles of 1min, at near 0°C temperature), n = 3, mean±SD.

Pressure (Mpa)	Cycles	Total protein (g/L)	Total IgG (mg/ml)	Total IgM (mg/ml)	IgG1 (mg/mL)	IgG2 (mg/mL)	IgG3 (mg/mL)	IgG4 (mg/mL)
**0**	**0**	48.9±1.17	4.2±1.35	0.9±0.12	3.5±0.74	3.5±0.06	0.57±0.04	0.13±0.00
**200**	**1**	50.0±0.27	7.3±0.10	1.0±0.06	4.4±0.79	2.7±1.06	1.2±0.52	0.1±0.02
	**2**	50.8±2.02	2.7±0.45	0.7±0.02	3.5±0.89	2.9±0.16	0.6±0.07	0.1±0.02
	**3**	52.1±0.43	4.0±0.55	0.7±0.01	3.1±0.06	2.3±0.16	0.3±0.03	0.1±0.01
	**4**	49.3±1.64	8.0±1.70	0.8±0.17	3.4±0.6	3.0±0.2	0.6±0.03	0.1±0.02
	**5**	49.6±0.77	6.2±1.65	0.8±0.12	3.5±0.28	3.3±0.48	0.6±0.03	0.1±0.01
**250**	**1**	47.5±2.63	7.4±3.75	0.7±0.08	3.2±1.25	1.8±0.59	0.9±0.39	0.1±0.01
	**2**	56.8±2.59	5.7±0.45	0.7±0.07	2.8±0.47	2.5±0.21	0.5±0.13	0.1±0.02
	**3**	44.8±3.23	7.7±1.85	1.2±0.55	3.4±0.85	2.8±0.37	0.6±0.01	0.1±0.01
	**4**	46.4±3.28	4.6±3.30	0.6±0.03	2.9±0.15	3.0±0.24	0.5±0.07	0.1±0.01
	**5**	53.6±0.98	3.8±0.65	1.0±0.10	2.5±0.06	2.7±0.02	0.5±0.00	0.1±0.00

There are some limitations in our study. The volume of sample is 5 mL, for the practical application, evaluation in larger sample volumes is required. Whether the increase of volume would change the sensitivity of plasma protein or inactivation efficacy under HHP requires further investigation. Furthermore, in our study, the content of plasma proteins were not affected by HHP, but the change of molecular structure is yet to be answered. Previous reports found at higher pressure level (>400MPa), most proteins tend to unfold, and the unfolded state remains considerably structured [31]. Presumably, in our test condition (200-250MPa), the plastic deformation of proteins is less intensive than higher pressure.

## Conclusion

After evaluating the activities of most plasma proteins and the inactivation efficacy of several blood-borne pathogens, the results of our study indicate that a narrow range of pressures and a lower temperature is useful for HHP in plasma.
